# Transformations of Selected *Fusarium* Toxins and Their Modified Forms During Malt Loaf Production

**DOI:** 10.3390/toxins12060385

**Published:** 2020-06-11

**Authors:** Marcin Bryła, Edyta Ksieniewicz-Woźniak, Agnieszka Waśkiewicz, Tomoya Yoshinari, Krystyna Szymczyk, Grażyna Podolska, Romuald Gwiazdowski, Krzysztof Kubiak

**Affiliations:** 1 Department of Food Analysis, Prof. Waclaw Dabrowski Institute of Agricultural and Food Biotechnology, Rakowiecka 36, 02-532 Warsaw, Poland; edyta.wozniak@ibprs.pl (E.K.-W.); krystyna.szymczyk@ibprs.pl (K.S.); 2 Department of Chemistry, Poznan University of Life Sciences, Wojska Polskiego 75, 60-625 Poznan, Poland; agnieszka.waskiewicz@up.poznan.pl; 3 Division of Microbiology, National Institute of Health Sciences, 3-25-26 Tonomachi, Kawasaki-ku, Kawasaki-shi, Kanagawa 210-9501, Japan; t-yoshinari@nihs.go.jp; 4 Department of Cereal Crop Production, Institute of Soil Science and Plant Cultivation—State Research Institute, Czartoryskich 8, 24-100 Pulawy, Poland; aga@iung.pulawy.pl; 5 Department of Pesticide Investigation, Institute of Plant Protection—National Research Institute, Wladysława Wegorka 20, 60-318 Poznan, Poland; R.Gwiazdowski@iorpib.poznan.pl (R.G.); K.Kubiak@iorpib.poznan.pl (K.K.)

**Keywords:** baking, malt, bread, *Fusarium* toxins, trichothecenes, zearalenone, modified mycotoxins

## Abstract

An increasing number of studies have found that modified mycotoxins, such as free mycotoxins, naturally occur in food, and severely impact food safety. The present study investigated concentrations of trichothecenes nivalenol (NIV), deoxynivalenol (DON), and zearalenone (ZEN), together with their modified forms, nivalenol-3-glucoside (NIV-3G), deoxynivalenol-3-glucoside (DON-3G), and zearalenone-14-glucoside (ZEN-14G) and zearalenone-14-sulfate (ZEN-14S), respectively, at successive stages of malt loaf production (flour, dough kneading/fermentation, loaf baking). Toxins in bakery products originate in flour produced from wheat grain that is naturally contaminated with *Fusarium culmorum*. Mycotoxin concentrations were determined using high-performance liquid chromatography-high resolution mass spectrometry, and did not significantly change during the successive stages of bread production. After the dough kneading/fermentation stage, concentrations of NIV-3G and DON-3G were slightly increased, whereas those of ZEN and ZEN-14S were slightly decreased. The largest average decrease (21%) was found in ZEN-14G. After the baking stage, the average concentrations of NIV-3G, DON-3G, ZEN-14S, and ZEN-14G in the loaf crumb and crust decreased by 23%, 28%, 27%, and 20%, respectively, compared with those in the dough. During this technical process, the concentration of ZEN-14G in loaf crumb significantly decreased by an average of 48%, and those of ZEN, ZEN-14S, and ZEN-14G in loaf crust decreased by an average of 29%, 42%, and 48%, respectively. Considering the possibility of modified mycotoxins degradation to free forms, as well as the ability to synthesize them from free forms during technological processes, it would be prudent to consider them together during analysis.

## 1. Introduction

Wheat, a basic commodity used in the baking industry, is vulnerable to *Fusarium* head blight (FHB), one of the most serious diseases of various field crops [1]. Many *Fusarium* species can cause FHB, but the most prevalent are *F. graminearum* and *F. culmorum* [2,3,4]. These fungi not only cause significant crop losses, but also deposit toxic metabolites (mycotoxins) in grains, which might cause harm to consumers [5,6]. Among the more than 400 known mycotoxins synthesised by various fungal species, those produced by *Fusarium* are probably the most hazardous in terms of food safety. *Fusarium* mycotoxins include several trichothecenes, including deoxynivalenol (DON), nivalenol (NIV), HT-2 toxin, T-2 toxin, and zearalenone (ZEN) [7,8]. Trichothecenes are among the most frequent toxins in cereal grains. They are not classified by the International Agency for Research on Cancer as carcinogenic to humans [9]. However, they can cause unfavourable health effects, such as inflammation of the gastric and intestinal mucosa, vomiting, weakening of the immune system, skin inflammation, and bleeding [10,11]. Studies from recent years have shown higher human exposure to DON than the tolerable daily intake (TDI). [12,13]. In addition, the prevalence of DON, which is associated with hyperestrogenism syndrome and haematotoxicity, is higher than that of ZEN, which might disrupt reproductive functions in animals, and has been detected in various cereal-based foodstuffs worldwide [14,15,16].

Modified mycotoxins might naturally occur in grains, and, like their basic analogues, might seriously impact the safety of cereal-based foods. Modified mycotoxins, such as deoxynivalenol-3-glucoside (DON-3G), nivalenol-3-glucoside (NIV-3G), and zearalenone-14-glucoside (ZEN-14G), can result from metabolic reactions in infected plants, or, like the mycotoxin ZEN-14S, can be directly produced by fungi [16,17,18]. Therefore, many aspects of modified mycotoxins, such as their toxicity, natural occurrence profiles, and stability during processing have been extensively studied.

The entire baking industry is based on cereal-based products. Malts are frequently added to various flours to improve their fermentation abilities, and the organoleptic properties of bread and/or can be added as dietary supplements, such as B group vitamins and minerals [19,20]. Up to 50% of malt flours used in the production of malt breads deliver flavourful and delicious products, which are immensely popular in the UK. Pale malts added to dough are enzymatically active, resolving starch into glucose and partially decomposing gluten [21]. Numerous malt active enzymes help to keep CO_2_ within the dough, by influencing the rheological properties of gluten bound with other flour constituents. Malt enzymes can change the chemical structure of mycotoxins, which consequently modifies their toxicity. The baking industry commonly also adds bread improvers, such as enzymes, to flour to improve and stabilise the quality [22]. These enzymes can also change the chemical structure of mycotoxins during dough fermentation. Besides, baker’s yeast (*Saccharomyces cerevisiae*) and lactic acid bacteria (LAB), used in dough fermentation produce numerous enzymes with the potential to modify mycotoxins [23,24].

The concentrations of modified mycotoxins are higher in malts than in cereal grains, sometimes even exceeding those of free mycotoxins [25,26]. Therefore, the stability of free and modified mycotoxins during bread production should be investigated in detail in terms of food safety. The published data are ambiguous. For example, DON concentrations were found to decrease in one study [25] and increase in other studies [27,28,29] during dough fermentation. Similarly, DON concentrations have been found to decrease in some studies [28,30], but remain highly stable in others [25,31] during loaf baking. Similarly, the results are contradictory with respect to the stability of DON-3G during loaf baking [25,27,32,33,34]. The stability of DON and DON-3G might depend on the temperature of dough fermentation that would influence the activities of enzymes in the dough [35]. Contrary to DON and DON-3G, data regarding the stability of NIV, NIV-3G, and ZEN are very scarce, and even more inconclusive. The concentrations of ZEN decrease during baking in bread prepared from naturally contaminated flour [36,37,38], and remain stable during fermentation with *S. cerevisiae* and baking [39]. Information on the stability of ZEN derivatives is not available.

As far as we can ascertain, this report describes the first study on the stability of DON, NIV, ZEN, and their modified derivatives during successive stages of bread production (flour, dough kneading/fermentation, loaf baking). To our knowledge, this is the first study to reveal novel findings about modified mycotoxins.

## 2. Results and Discussion

Mycotoxins and their modified forms were determined using a validated method. With respect to DON and ZEN, the method recovery rate (R) and precision (relative standard deviations (RSD)) can be evaluated according to the performance criteria specified within Commission Regulation (EC) No 401/2006. Since no criteria are available regarding modified forms of DON and ZEN, we applied the criteria concerning their free forms. Likewise, no criteria are available for NIV and NIV-3G, but since they are trichothecenes that structurally resemble DON and DON-3G, we applied the criteria for DON and DON-3G. The EU Regulation specifies that a method should perform well at fortification levels > 50 and > 500 µg/kg for ZEN and DON, respectively; recovery rates should fall within 70–120%, and the method precision should be ≤25% for ZEN and ≤20% for DON. Our analytical method met these criteria and was therefore regarded as being suitable.

Malt flour can be naturally contaminated with high levels of *Fusarium* mycotoxins and their metabolites. The Commission Regulation (EC) 1881/2006 [40] specifies that contamination with DON and ZEN at concentrations > 750 and > 75 µg/kg, respectively, would exclude flour from human consumption. According to Commission Regulation (EC) 178/2002 [41], raw material contaminated like this must not be dissolved before use. However, we used contaminated flour here, to identify the transformations of modified mycotoxins during malt loaf production.

### 2.1. Trichothecenes and Their Modified Forms

The malt flour used herein was highly contaminated with mycotoxins and their modified forms. To reveal the transformation and/or degradation of mycotoxins during bread production, the results obtained from all samples were normalised to the amount of flour used to produce them. Mean mycotoxin concentrations in samples collected at successive stages of bread production (flour, fresh dough, dough after 1st fermentation, ready-to-bake dough after 2nd fermentation, loaf crumb, loaf crust) were normalised to the amount of flour used to produce the samples, considering the water and dough additives (yeast, salt, sugar) described in Table S1. 

The concentrations of NIV, DON and NIV-3G in the flour were 330 ± 21, 1065 ± 138 µg/kg, (887 ± 116 µg/kg, the latter being over twice that of NIV. The concentration of DON-3G (601 ± 102 µg/kg) was almost twice as low as that of DON. Neither NIV nor DON concentrations significantly changed at subsequent stages of malt loaf production, according to analyses of variance (ANOVA).

**Figure 1:**
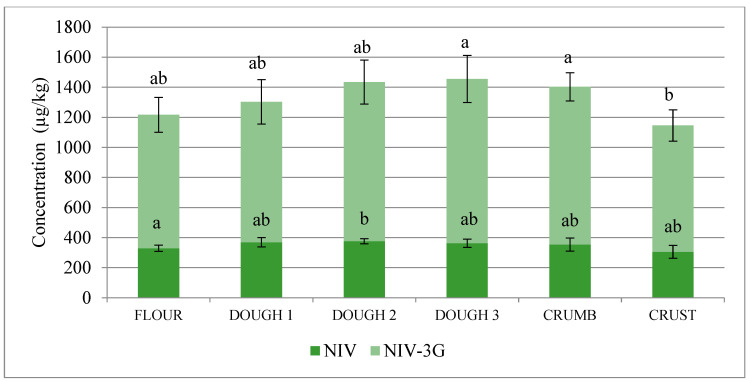
Combined nivalenol (NIV)+nivalenol-3-glucoside (NIV-3G) concentrations at successive stages of malt loaf production (flour, fresh dough 1, dough 2 after the 1st fermentation, ready-to-bake dough 3 after the 2nd fermentation, loaf crumb, and loaf crest). Data are normalised to the amount of flour used to produce each sample, considering water and dough additives (yeast, salt, sugar). Different letters mark different homologous groups (significance level α = 0.05).

**Figure 2:**
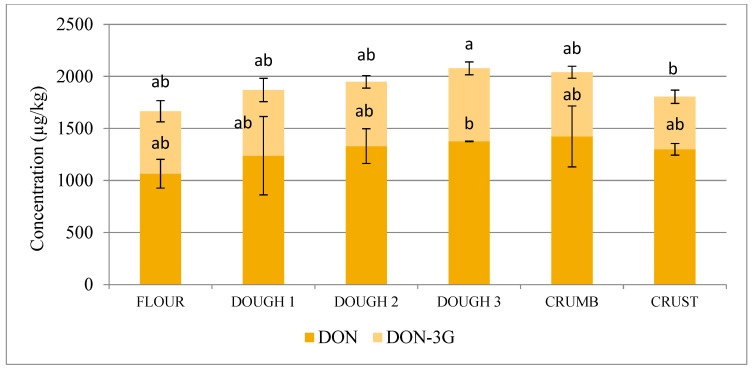
Concentration of combined deoxynivalenol (DON)+deoxynivalenol-3-glucoside (DON-3G) at successive stages of malt loaf production (flour, fresh dough 1, dough 2 after the 1st fermentation, ready-to-bake dough 3 after the 2nd fermentation, loaf crumb, and loaf crest). Data normalised to the amount of flour used to produce each given sample considering water and dough additives (yeast, salt, sugar). Different letters mark different homologous groups (significance level α = 0.05).

Combined concentrations of trichothecenes and their glucosides increased during the flour, dough 1, dough 2, and dough 3 stages of bread production (1217, 1303, 1434, and 1455 µg/kg, respectively, for NIV+NIV-3G and 1666, 1869, 1948, and 2078 µg/kg, respectively, for DON+DON-3G) and decreased after baking the loaf (down to 1146 µg/kg of NIV+NIV-3G in loaf crest and down to 1805 µg/kg of DON+DON-3G in loaf crest), see Figure 1 and Figure 2.

Table 1 shows that the molar ratios of NIV-3G:NIV, DON-3G:DON, ZEN-14S:ZEN, and ZEN-14G:ZEN changed slightly during the successive stages of bread production. For example, at the flour, dough 1, dough 2, and dough 3 stages, the NIV-3G:NIV ratios were 1.77, 1.66, 1.85, and 1.98, respectively, and the DON-3G:DON ratios were 0.36, 0.33, 0.30, and 0.33, respectively. Baking caused the ratio of NIV-3G:NIV to decrease to 1.95 and 1.80 in crumb and crust, respectively, and that of DON-3G:DON to decrease to 0.28 and 0.25 in crumb and crust, respectively. The larger decrease in crust suggests higher susceptibility to glucosides than free mycotoxins, due to thermal degradation.

Temperature is important, but it is not the only factor that influences reactions of degrading, transforming, linking, and releasing mycotoxins during the bread production processes. Other factors include bread improvers, malts, lactic acid bacteria, and baker’s yeast.

Our results are difficult to compare with published data because other researchers usually did not consider the changes in mycotoxin concentrations resulting from changes in the moisture content and composition of their samples. However, few reports have described the transformation of modified trichothecenes and ZEN during bread production. The most prevalent enzymes added to improve bread taste and nutritional value are α-amylase, xylanase, and sometimes protease [42]. These water-activated enzymes are also found in cereals and cereal products, including various flours and malts [43]. Kostelanska et al. [44] were among the first to assess the influence of enzymes in bread improvers on the transformation of DON and DON-3G during bread production. They found that in bread baked with and without improvers, DON levels increased by an average of ~32% and ~14%, respectively, whereas DON-3G levels decreased by ~32% and ~50%, respectively.

Enzymes produced by live cereal plants might increase the levels of DON by liberating it from the cell walls of grains and other plant cells. Simsek et al. [31] incubated ground wheat naturally contaminated with DON, in buffers at 40–60 °C. They found that DON levels increased by 16% and 39% when protease was added at pH 7.0 and 50 °C and xylanase was added at pH 4.7 and 40 °C, respectively. The findings of cellulase (pH 4.7, 45 °C) were inconclusive, because although DON levels increased by 25%, there were comparable increases without added enzyme. Other enzymes (α-amylase at pH 6.5, 60 °C) did not influence the DON levels. The researchers also reported that DON-3G concentrations remained stable during dough fermentation, and decreased after bread baking, indicating that DON-3G is less thermally stable than DON.

Changes in trichothecene concentrations during bread production herein were less significant than those reported by Simsek et al. [31] and Kostelanska et al. 2011 [44]. One explanation might be that we used naturally contaminated malt flour, whereas these studies included bread baked with some improvers. Malt is produced by soaking and germinating grain, which starts life processes, during which enzymes might liberate mycotoxins from grain cell walls or other constituents. Vidal et al. [35] investigated the influences of xylanase, α-amylase, cellulase, protease, lipase, and glucose oxidase on DON concentrations at dough fermentation temperatures of 30 °C and 45 °C. That study found that only α-amylase activity increased, resulting in slightly higher concentrations of DON, compared with controls (without added enzymes). Concentrations of DON were higher if the bread was baked from dough with added xylanase or α-amylase. Concentrations of DON were higher if any one of xylanase, cellulase, protease, lipase, or glucose oxidase was added. On the other hand, the DON-3G concentration increased in dough fermented without added enzymes. After baking, it decreased to about 80%, which was 50% of the DON-3G level in the ready-to-bake dough, under both sets of conditions described by Vidal et al. [35]. In another study, Vidal et al. [45] baked bread from a flour with some added α-amylase and xylanase, and found that that DON concentrations in the bread were higher compared with bread without added enzymes.

The pH steadily decreases during successive stages of bread production and is 5.76, 5.42, 5.37, and 5.27 in flour, ready-to-bake dough, loaf crumb, and loaf crust, respectively (Table 1). The fermentation environment becomes more acidic if the process is assisted by either *S. cerevisiae* yeast (used in this study) or LAB [46]. Changes in pH mean that acidity becomes sub-optimal for various enzymes. The pH within any dough fermentation environment is a likely essential factor that influences the rates at which mycotoxins in dough are transformed at various temperatures. According to some reports, mycotoxins (including DON) are structurally less stable in alkaline environments [47,48,49], whereas others have found that mycotoxins are more prone to transform or degrade in more acidic environments [50].

Changes in DON levels in dough following fermentation are unclear. These levels have been reported to decrease [51,52], increase [53,54], or remain unchanged [28,55]. Levels might depend on less controlled factors (dough components, additives, initial concentration of the mycotoxin, species of LAB bacteria or baker’s yeast), in addition to the usually well-controlled duration of the process and process temperature. Our dough was fermented at 30 °C for 2 h. Samar et al. [56] reported different changes in DON concentration during dough fermentation with *S. cerevisiae* yeast (without bread improver), according to fermentation temperature and duration: after 60 min at 30 °C, the DON concentration remained essentially constant, after 40 min at 50 °C, it decreased by a maximum of 41%, after 60 min at 50 °C it decreased by a maximum of 56%. Vidal et al. [35] reported a 5% decrease in DON concentration during dough fermentation (without bread improver) at 30 °C, and a 23% decrease at 45 °C. Both DON-3G concentrations fell below the limits of quantification (LOQ). Ragab et al. [57] reported a 35% decrease in DON concentrations after fermentation at 32 °C for ~90 min. They suggested that the enzymes in flour or those produced by endogenous microorganisms were responsible for this, rather than the baker’s yeast. Neira et al. [30] showed a reduction in DON concentration by about 22% after yeast fermentation at 25 °C for 11 h.

The hypothesis that the influence of dough fermentation conditions on DON concentrations depends on its initial concentration was verified by Neira and co-workers [30], who found a positive correlation between DON initial concentration in flour and its reduction after dough fermentation. On the other hand, Dropa et al. [58] reported only slight (4–12%) decreases in DON levels after yeast fermenting wheat-based dough at 30 °C, regardless of the initial DON concentration (400–1600 µg/kg), regardless of the fermentation duration. 

Fermentation in the present study was assisted by yeast. However, flour microflora should also be considered when discussing mycotoxin transformations. Banu et al. [52] reported a ~35% decrease in DON concentrations after yeast fermentation (three *Lactobacillus* varieties, 28 °C, 30 min), compared with the initial concentration in flour, and a ~16% decrease after spontaneous fermentation (without starters). The researchers suggested that dough pH was the main factor responsible for the differences, since the pH of dough fermented without starters was 5.44, compared with 3.84–3.97 for dough with LAB bacteria. The researchers also reported a 59–67% reduction in DON levels by leavening prepared with some starters, compared with a 26–29% reduction in leavening made from the same flour that spontaneously fermented at 37 °C for 20 h.

The DON-3G and NIV-3G concentrations found herein were quite stable during dough kneading and fermentation. During baking, DON-3G was less thermally stable than DON (lower concentration in loaf crest). Generotti et al. [32] studied DON levels during yeast fermentation of dough suitable for whole wheat rusks. They reported the practical stability of DON and DON-3G levels at various fermentation conditions (temperature 26°C, 36°C, and 46 °C; duration 40 min, 50 min, and 60 min; with enzymes or without additives). Valle-Algarra et al. [59] reported similar results of DON and NIV stability during yeast fermentation for 1 h at 30 °C. De Angelis et al. [60] found a ~70% decrease in the concentration of DON-3G, whereas that of DON slightly increased during the entire process of bread production. Zhang and Wang [29] also reported that DON and DON-3G concentrations during yeast fermentation (30 °C, 150 min) concentrations increased by ~44%, but decreased by ~20% compared with initial levels in flour. The present finding that trichothecene concentrations increased at the dough kneading or fermentation stages (Table 2) was probably related to the fact that we started from malt contaminated with mycotoxin. Enzymes produced during malting might liberate DON from plant cell walls, but might also help to convert DON into DON-3G or to hydrolyse DON-3G into DON. A recent study has shown that DON-3G might be converted into DON during the steam processing of Chinese bread made of enzymatically deactivated wheat flour without yeast enzymes [61]. Mycotoxin molecules might be mechanically destroyed by friction forces at the dough kneading stage.

That the stability of mycotoxins might be disturbed mostly at the baking stage of the bread production when temperatures are much higher than at the earlier stages is to be expected. Thermal stability depends on the matrix, in which the mycotoxin exists (grain, processed food etc.); some matrix components might stabilise the molecular structure of mycotoxins [31]. We did not find any significant changes in either NIV or DON concentrations in either loaf crumb or crest compared with levels in ready-to-bake dough; a decrease in the concentrations of NIV (by 16%) and DON (by 6%) in loaf crest can be regarded as small. Additionally, the decrease in NIV-3G and DON-3G levels in loaf crumb was insignificant. On the other hand, NIV-3G and DON-3G decreased in loaf crest by 23% and 28%, respectively (Table 2). This may reflect the fact that crumb temperature usually does not exceed 100 °C, regardless of the baking temperature, due to the transport of water from inside the loaf towards its surface. On the other hand, loaf crest temperature might be much higher, which could result in decreased mycotoxin stability in the crest. Similar stability of DON during baking was reported by Stadler et al. [62], Simsek et al. [31], Vidal et al. [34], and Bergamini et al. [28]. Lancova et al. [51] reported a 26–31% reduction in DON levels during baking, but this became ~6% after normalisation of the results to the amount flour used. These conclusions concerning thermal stability of DON in loaf crest are in line with those of Numanoglu et al. [38].

### 2.2. ZEN and Its Modified Forms

Initial ZEN, ZEN-14S, and ZEN-14G concentrations in the flour were 1378 ± 153, 622 ± 182, and 407 ± 61 µg/kg, respectively (Table S1). The concentration of ZEN decreased to 1042 ± 128 µg/kg after the 1st fermentation (dough 2); in crest of the baked loaf it was only 984 ± 20 µg/kg, namely 28.6% less the than the initial concentration in the flour (Table 2). The ZEN-14S was also quite stable at the early stages of bread production, but its final concentration in the loaf crest was ~42% lower than the initial concentration in the flour. The ZEN-14G concentration steadily decreased from 407 ± 61 in the flour to 332 ± 15, 261 ± 16, 263 ± 6, 212 ± 6, and 210 ± 6 µg/kg in fresh dough 1, dough 2 after the 1st fermentation, ready-to-bake dough 3, loaf crumb, and loaf crest, respectively, totalling 48% compared with the flour. The ZEN-14S: ZEN molar ratios were also quite stable during the above stages (0.36, 0.29, 0.36, 0.34, 0.33, and 0.37, respectively), whereas the ZEN-14G:ZEN molar ratio steadily decreased during these stages (0.20, 0.18, 0.17, 0.16, 0.12, and 0.14, respectively). Thus, ZEN-14G seems less stable than ZEN-14S.

**Figure 3:**
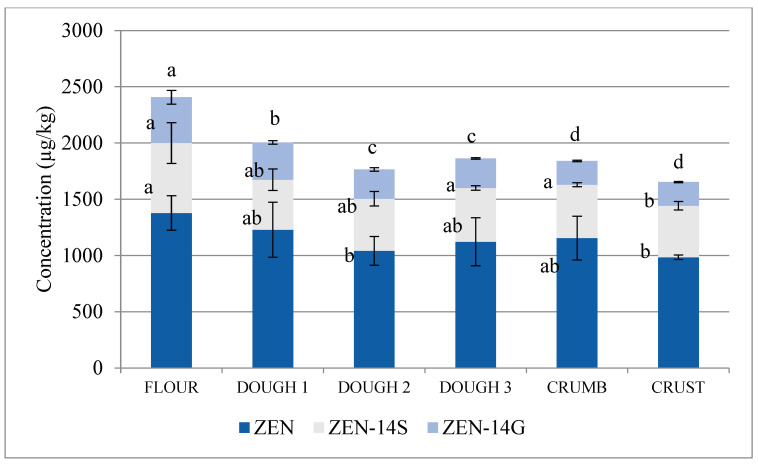
Concentration of combined ZEN+ZEN-14S+ZEN14G at successive stages of malt loaf production (flour, fresh dough 1, dough 2 after the 1st fermentation, ready-to-bake dough 3 after the 2nd fermentation, loaf crumb, and loaf crest). Data normalised to the amount of flour used to produce each given sample considering water and dough additives (yeast, salt, sugar). Different letters mark different homologous groups (significance level α = 0.05).

Contrary to trichothecenes, concentrations of combined ZEN, ZEN-14S and ZEN-14G almost steadily decreased from 2407 µg/kg in the flour to 1,863 µg/kg in the ready-to-bake dough 3, to 1841 µg/kg in the baked loaf crumb (22.4% compared with flour), and to 1653 µg/kg in the loaf crest (35.5% compared with flour) (Figure 3).

Unlike trichothecenes, ZEN concentrations decreased at almost all stages of bread production so the combined decreased from flour to baked loaf was ~29% (Table 2). However, no significant changes were evident between ready-to-bake dough and loaf crumb or loaf crust. Changes in the ZEN-14S concentration were similar. Combined ZEN-14G was relatively least stable. The trend in concentrations of the combined ZEN, ZEN-14S, and ZEN-14G suggest unknown directions of their transformations. Less is known about the behaviour of ZEN than DON and its derivatives during bread production. Besides, most of these studies did not investigate the intermediate stages of the process (dough kneading, fermentation). Matsuura et al. [36] reported a decrease in the ZEN concentration of 31–40% during loaf baking at 190–200 °C for 30 min; fermentation did not seem to significantly impact the reduction. A similar decrease (12–43%) was found after baking of wheat bread from ZEN-fortified flour (200 °C, 20 min). Heidari et al. [63] reported a decrease in the ZEN concentration during bread production. Four variants of fermentation were studied: active dried yeast, instant yeast, LAB bacteria, and pressed yeast, and the decreases amounted to 36%, 39%, 40%, and 68%, respectively. Cano-Sancho et al. [39] reported a 35% drop in the ZEN concentration after baking wheat bread at 200 °C for 20 min; the researchers probably did not consider dilution effects and constituents other than flour dough. Numanoglu et al. [38] reported a 13% decrease in the ZEN concentration while baking corn bread at 250° for 70 min. On the other hand, Bol et al. [64] reported an 89% decrease in ZEN concentration after baking wheat bread, which is a conspicuously high value. The researchers normalised the ZEN concentration to dry mass, but did not consider the dilution effects. Even a high ratio of flour in bread (95%) cannot explain such a sharp decrease in the ZEN concentration.

## 3. Conclusions

The concentrations of the studied mycotoxins did not change much during malt bread production (dough kneading, dough fermentation, loaf baking). However, the changes differed among mycotoxins. The concentrations of trichothecenes and their modified forms (NIV-3G and DON-3G) increased during dough kneading and fermentation, most likely as a result of the activities of the enzymes in the malt flour. In contrast, concentrations of ZEN and ZEN-14S slightly fell during these stages of the process. A significant average decrease of 21% was found only for ZEN-14G. Changes from ready-to-bake dough to baked loaf crumb were insignificant, except for ZEN-14G, the concentration of which decreased by 19%. However, the average concentration decreases from ready-to-bake dough to baked loaf crest were significant in a few studied mycotoxins: 23%, 28%, 27%, and 20%, respectively, for NIV-3G, DON-3G, ZEN-14S, and ZEN-14G. Since the concentration of ZEN, combined with its derivatives, decreased during the malt bread production, these mycotoxins might have been transformed into compounds with different structures. Enzymes present in malt flour probably facilitate the deconjugation or hydrolysis of modified mycotoxins. In the absence of a clear consensus among scientists about the toxicity of modified mycotoxins [65,66], it must be assumed that they have comparable toxicity to their free forms. During bread production, they can be synthesised from free forms and can be degraded into free forms. Therefore, it would be prudent to consider them jointly in the determination of mycotoxins. The goal of this study and related studies is to prevent unfavourable health effects in consumers exposed to foods that are not routinely tested for mycotoxins and their modified forms.

## 4. Materials and Methods

### 4.1. Chemicals and Reagents

We purchased certified analytical DON, NIV (100 µg/mL in acetonitrile), DON-3G (50.2 µg/mL in 50:50 *v/v* acetonitrile:water), and ZEN (101.0 µg/mL in acetonitrile) standards from Romer Labs Division Holding GmbH. (Tulln an der Donau, Austria). We obtained certified analytical ZEN-14G (10 µg/mL in 1/1 *v/v* acetonitrile:water) and ZEN-14S (97.7 µg/mL in acetonitrile) standards from Aokin AG. (Berlin, Germany). We isolated NIV-3G (110 µg/mL) from wheat as described by Yoshinari et al. [67]. Solutions stored at −20 °C were brought to room temperature (~20 °C) before use. The following reagents: HPLC-grade acetonitrile, HPLC- and LCMS-grade methanol, water, 98–100% formic acid, ammonium formate, and LCMS-grade formic acid were purchased from Witko Sp. z o. o. (Łódź, Poland).

### 4.2. Samples

Malt bread was prepared using type 750 whole-bread wheat flour that did not contain any of the known mycotoxins, and malt flour that was naturally contaminated with the mycotoxins. The latter was prepared using malt produced at a micromalting facility operated at the Prof. Wacław Dąbrowski Institute of Agriculture and Food Biotechnology (Warsaw, Poland) from the *Legenda* genotype wheat grain (whole bread variety). 

Grains cultivated on test plots run by the Institute of Plant Protection - National Research Institute (Poznań, Poland) were inoculated with *F. culmorum* strain KZF-5 from the collection at the Research Centre for Registration of Agrochemicals of the Institute of Plant Protection - National Research Institute (Poznan, Poland). Plants were inoculated on May 29, 2018 (vegetation season), at the BBCH 61 growth stage (beginning of flowering). An aqueous suspension of spores (0.5 × 10^6^/mL) was applied to the plots, using a knapsack sprayer, at a pressure of 0.2 bar and a water volume of 300 L/ha. The grains were then harvested using a Wintersteiger plot harvester on August 2, 2018. 

The grains were malted according to the Mitteleuropäische Brautechnische Analysenkommission instructions 1.5.3 ed. 2011, with some modifications in the soaking procedure and the duration of sprouting. The grains (~1 kg) were placed in a container with a perforated bottom, maintained at 14 °C and relative humidity > 95%. The grains were soaked for 7 h, aerated for 17 h, and soaked again for 7 h in fresh water. They were then sprinkled with water to increase the moisture level by up to 44% by weight. The grain was mixed daily during a sprouting period of 7 days. The malt obtained after 7 days was dried for 24 h, then placed at ~45 °C for about 18 h. During this preliminary drying phase, the malt humidity fell below 10%, which limited microbial metabolic functions and decreased enzyme reaction rates. The temperature of the dryer unit was gradually increased to 80 °C within 1 h, and held for 4 h (final drying). After cooling, the malt was pulverised for 3 min in a Grindomix GM200 (Retsch GmbH., Haan, Germany) mill at 1000 revolutions per second (rps), and passed through a 0.23 mm round mesh sieve. Bread was then prepared using the sifted malt flour.

### 4.3. Laboratory Baking

Type 750 whole bread wheat flour was mixed together with a malt flour (1:1) in a V10L (Stalgast Sp. z o. o, Warsaw, Poland) spiral mixer at 15 rpm for 2 h. The flour mixture (540 g) was combined with water (300 mL), *S. cerevisiae* baker’s yeast (18 g), salt (9 g), and sucrose (6 g). The leaven used to make the dough was prepared as follows. Fifteen grams of the flour and 75 mL of water with added yeast and sugar were mixed and put aside to rise for 15 min at 30 °C. Next, the remaining constituents were added, and the dough was mixed in the spiral mixer, until it could be freely separated from the dish walls (for about 20 min). The dough was left to rise in a BC G-100/250 heater (Premed, Warsaw, Poland) at 30 °C. After 1 h, ~20 g dough pieces were formed, and fermentation was continued for 1 h. The risen dough pieces were baked in a KS-70S50BSS (Sharp Corp., Osaka, Japan) oven for 25 min at 230 °C. Three baking series were run with four pieces of dough baked in each series. The bread was cooled to room temperature and weighed. Loaf crumb was separated from the crest and the amounts of both in the bread were determined. Samples taken from every stage in the bread production process (flour, fresh dough, dough after the 1st fermentation, ready-to-bake dough after the 2^nd^ fermentation, crest, and crumb) were placed at –30 °C for 24 h, then lyophilised in an ALPHA 1-4 LSCplus (Martin Christ Gefriertrocknungsanlagen GmbH., Osterode am Harz, Germany) unit for 16 h at 25 °C. Dried samples were weighed and milled in a Grindomix GM200 mill (Retsch GmbH.) for 1 min at 1000 rps.

### 4.4. Sample Preparation

Acidity levels were measured in samples of flour, fresh dough, dough after the 1st fermentation, ready-to-bake dough, loaf crumb, and loaf crest. Each homogenised sample was mixed with deionised water (1:2) and measured in triplicate using a CPC-505 calibrated pH metre (Elmetron Sp. J., Zabrze, Poland).

Analytical samples were prepared as described by Nathanail et al. [68], with some modifications (“dilute and shoot” approach). Sample (0.5 g) and NaCl (0.1 g) were homogenised in 2 mL of extraction liquid (20:79:1 v/v/v acetonitrile:water:acetic acid), in a MM 400 ball mill (Retsch GmbH.) at 30 rps for 4 min. The homogenate was passed through a 0.45-μm syringe filter, then 350 μL of the filtrate was evaporated in a stream of nitrogen. The residues were dissolved in 700 μL of 50% methanol, and passed through a 0.22-μm. Filtrates were analysed in triplicate.

### 4.5. Liquid Chromatography-Mass Spectrometry (LC-TOF-HRMS)

Samples were analysed using an H-class liquid chromatograph, coupled with a UPLC-time-of-flight high-resolution mass spectrometer (TOF-HRMS) (Waters Corp., Milford, MA, USA). Analytes were separated on a UPLC C18 Cortecs column (2.1 × 100 mm, 1.6 μm), equipped with a pre-column (Waters Corp). A total of 0.2% formic acid and 50 mM ammonium formate were added to the mobile phase A (90:10 *v/v* methanol:water) and phase B (10:90 *v/v* methanol:water). The phases were passed through the column at a flow rate of 0.3 mL/min as follows: 100% B 0–2 min; 50% B 3–6 min; 100% A 22–23 min; 100% B for 25–28 min. The mass spectrometer was operated in the negative ionisation mode with electrospray and ion source, with desolvation temperatures of 150 °C and 350 °C, respectively. The flow rates of the nebulising (N_2_) and drying gases were 650 and 40 L/min, respectively. Capillary bias was 2.2 kV. Ion optics were operated in the W mode. The instrument was calibrated using a leucine-enkephalin solution.

### 4.6. Statistical Analysis

Significant differences between concentrations of the detected mycotoxins were assessed by single factor analyses of variance (ANOVA) using the Statgraphics 4.1 (Statgraphics Technologies Inc., The Plains, VA, USA) software. Values with α = 0.05 significance were selected using the Fisher tests (LSD). Statistically homologous groups of results are shown with the same superscript letters.

### 4.7. Method Validation

Linearity range, limits of detection (LOD, S/N = 3), limits of quantitation (LOQ, S/N = 10), recovery rates (R%), and repeatability (RSD%) were determined. Calibration curves were separately prepared for flour and bread. Matrix-embedded standards were prepared from mycotoxin-free flour in the same manner as the test samples, except various amounts of analytical mycotoxin standards were added between the solvent evaporation step and final dissolution in 30% methanol. Calibration curves for analytes were within the following concentration ranges (μg/kg): NIV, 125–1000; NIV-3G, 50–396; DON, 250–10,000; DON-3G, 125–3500; ZEN, 34–2200; ZEN-14G and ZEN-14S, 68–2200. Correlation coefficients in 13 of 14 cases were > 0.985, and the ranges of LOD and LOQ values were respectively, 10–75, and 34–250 µg/kg (Table 3).

Method recovery rates (R) and repeatability (RSD) were determined in samples of mycotoxin-free flour fortified with three concentrations of analytical standards. Ranges of fortification depending on the matrix and analyte were 50–2500, 100–5000, and 200–10,000 µg/kg. These, and the test samples, were analysed in triplicate. The fortified samples were analysed at every analytical run to confirm recovery rates ranging from 74–111.1%, depending on the fortification level and the analyte. The RSD never exceeded 20% (Table 4).

## Figures and Tables

**Table 1:** Molar ratios of mycotoxins in samples collected at successive stages of malt loaf production (flour, fresh dough 1, dough 2 after the 1st fermentation, ready-to-bake dough 3, loaf crumb, loaf crust).

Molar Ratio	Flourp H 5.76 ± 0.09 (n = 3)	Dough 1 pH 5.72 ± 0.10 (n = 3)	Dough 2 pH 5.59 ± 0.08 (n = 3)	Dough 3 pH 5.42 ± 0.12 (n = 3)	Crumb pH 5.37 ± 0.10 (n = 12)	Crust pH 5.27 ± 0.11 (n = 12)
NIV-3G/NIV	1.77	1.66	1.85	1.98	1.95	1.80
DON-3G/DON	0.36	0.33	0.30	0.33	0.28	0.25
ZEN-14S/ZEN	0.36	0.29	0.36	0.35	0.33	0.37
ZEN-14G/ZEN	0.20	0.18	0.17	0.16	0.12	0.14

**Table 2:** Mean change (%) in concentrations of mycotoxins between subsequent stages of bread production (left) and during entire process (right).

Analyte	Kneading	Fermentation		Baking		Whole Process	
	Dough 1/Flour	Dough 2/Dough 1	Dough 3/Dough 2	Crumb/Dough 3	Crust/Dough 3	Crumb/Flour	Crust/Flour
NIV	+12.1	+1.6	−3.5	−2.5	−15.7	+7.3	−7.3
NIV-3G	+5.3	+13.3	+3.2	−3.9	−23.2	+18.3 *	−5.4
NIV+NIV-3G	+7.1	+10.0	+1.5	−3.6	−21.3 *	+15.3	−5.9
DON	+16.2	+7.5	+3.4	+3.4	−5.5	+33.6 *	22.1 *
DON-3G	+5.0	−2.2	+13.8	−12.1	−28.1	+2.7	−16.0
DON+DON-3G	+12.2	+4.2	+6.7	−1.8	−13.1	+22.4 *	+8.3
ZEN	−10.7	−15.3	+7.7	+2.9	−12.3	−16.2	−28.6 *
ZEN-14S	−22.2 *	+0.4	+1.6	+1.4	−27.3	−19.5 *	−42.3 *
ZEN-14G	−18.4	−21.4	+0.8	−19.4	−20.2	−47.9	−48.4 *
ZEN+ZEN-14S+ZEN-14G	−15.0	−12.6	+5.0	−0.6	−17.3	−22.4 *	−35.5 *

* Statistically significant difference. Symbols − and + indicate mean concentration reduction and increase, respectively in concentrations.

**Table 3:** Parameters of HPLC/MS and limits of detection (LOD), limits of quantification (LOQ), and correlation coefficients of analytes.

Analyte	Ion Mass (m/z)	Retention Time (min)	LOD (μg/L)	LOQ (μg/L)	Coefficient of Determination (R^2^)	
					Flour	Bread
NIV	357.21 (M+FA-H)^−^	2.38	38	125	0.9155	0.9889
NIV-3G	519.22 (M+FA-H)^−^	2.45	15	50	0.9906	0.9858
DON	341.22 (M+FA-H)^−^	4.08	75	250	0.9892	0.9988
DON-3G	503.23 (M+FA-H)^−^	4.22	38	125	0.9969	0.9987
ZEN	317.10 (M-H)^−^	11.65	10	34	0.9958	0.9910
ZEN-14G	525.11 (M+FA-H)^−^	6.63	20	68	0.9981	0.9887
ZEN-14S	397.09 (M-H)^−^	7.25	20	68	0.9970	0.9941

LOD, limits of detection; LOQ, limits of quantitation.

**Table 4:** Relative standard deviations in % (RSD) and recovery in % (R) rates of mycotoxins at various fortification levels.

Mycotoxin	Bread			Flour		
	Fortification (μg/kg)	RSD (%)	R (%)	Fortification (μg/kg)	RSD (%)	R (%)
NIV	125	10.5	87.3	250	10.5	81.9
	250	3.5	76	500	13.9	90.9
	500	5.3	78	1000	19.2	94.1
NIV-3G	50	11.9	79.1	100	16.1	91.4
	100	15.2	99	200	8.1	98.2
	200	6.9	77	400	11.3	100
DON	1250	4.6	94.3	2500	4.8	99.9
	2500	5.2	84	5000	4.6	98.9
	5000	4.6	78	10,000	10.2	111.1
DON-3G	400	13.8	102	800	13.2	100.8
	800	15.6	74	1600	8.1	99.7
	1600	16.7	81	3200	11.2	109.7
ZEN	320	3.8	79.3	320	14.1	109
	630	4.1	81	630	6.4	97
	1260	4.5	87	1260	8.5	97
ZEN-14G	64	8.1	80	64	12.3	102
	126	4.3	83	126	8.2	111
	252	7	86	252	8.5	103
ZEN-14S	64	9.5	84.4	64	18.5	103
	126	7.1	80	126	16.3	109
	252	4.8	81	252	10.6	90

## Supplementary Materials

Supplementary Materials can be found at https://www.mdpi.com/2072-6651/12/6/385/s1.
