# Supplementary material for: Transformations of Selected Fusarium Toxins and Their Modified Forms During Malt Loaf Production
Source: Toxins (Basel). 2020 Jun 11;12(6):385. doi: 10.3390/toxins12060385 (PMC7354580; doi:10.3390/toxins12060385)
Supplement: Supplementary file 1 [file toxins-12-00385-s001.pdf]

Supplementary Materials: Transformations of Selected Fusarium Toxins and Their Modified Forms During Malt Loaf Production

Marcin Bryła, Edyta Ksieniewicz-Woźniak, Agnieszka Waśkiewicz, Tomoya Yoshinari, Krystyna Szymczyk, Grażyna Podolska, Romuald Gwiazdowski and Krzysztof Kubiak

Table S1. Mean concentrations (μg/kg) of mycotoxins found in samples collected at successive stages of malt loaf production normalised to the amount of flour used to produce each sample and considering dough additives (yeast, salt, sugar). Different letters mark different homologous groups (significance level α=0.05).

Analyte	Flour pH 5.76 ± 0.09 (n = 3)	Dough 1 pH 5.72 ± 0.10 (n = 3)	Dough 2 pH 5.59 ± 0.08 (n = 3)	Dough 3 pH 5.42 ± 0.12 (n = 3)	Crumb pH 5.37 ± 0.10 (n = 12)	Crust pH 5.27 ± 0.11 (n = 12)
NIV	330±21^{a}	370±31^{ab}	376±17^{b}	363±27^{ab}	354±43^{ab}	306±43^{ab}
NIV-3G	887±116^{ab}	934±148 ^{ab}	1058±146^{ab}	1092±157^{a}	1049±94^{a}	839±104^{b}
DON	1065±138^{ab}	1238±377^{ab}	1331±167^{ab}	1376±26^{b}	1423±293^{ab}	1300±56^{ab}
DON-3G	601±102^{ab}	631±112^{ab}	617±60^{ab}	702±62^{a}	617±58^{ab}	505±64^{b}
ZEN	1378±153^{a}	1230±245^{ab}	1042±128^{b}	1122±213^{ab}	1155±19^{ab}	984±20^{b}
ZEN-14S	622±182^{a}	484±96^{ab}	486±65^{ab}	494±20^{a}	501±18^{a}	359±37^{b}
ZEN-14G	407±61^{a}	332±15^{b}	261±16^{c}	263±6^{c}	212±6^{d}	210±6^{d}
